# Longevity, lipids and *C. elegans*

**DOI:** 10.18632/aging.100288

**Published:** 2011-02-24

**Authors:** A.J. Hulbert

**Affiliations:** School of Biological Sciences, University of Wollongong, Wollongong, NSW 2522, Australia

Scientific investigation of mechanisms that determine lifespan can be divided into three general approaches. The first approach (the comparative method) began over a century ago comparing species differing greatly in maximum longevity and implicated a role for the speed of metabolism in determining the length of life [1]. The second approach commenced after the 1930s investigated the mechanisms whereby “calorie-restriction” extended lifespan [2]. The third approach gained popularity in the 1990s and centered on genetic mutations that significantly extend longevity [3]. There has been little overlap between these three approaches. The paper by Shmookler Reis *et al.* in this issue [4] is significant in that it advances our understanding by combining the genetic mutants approach with recent insights from the first approach.

The recent insight from the comparative approach has been to link membrane fatty acid composition to maximum lifespan [5]. This link grew from the finding that membrane fatty acid composition varied systematically with body-size among mammals [6] and the suggestion this caused different cellular metabolic rates in mammals [7]. Membrane fatty acid was then also linked to maximum lifespan (MLSP) variation among mammals [8]. The reason why membrane fatty acid composition is correlated with MLSP is because fatty acids differ greatly is their susceptibility to lipid peroxidation. Only polyunsaturated fatty acids undergo peroxidation (saturates and monounsaturates are peroxidation-resistant), and the more polyunsaturated the fatty acid, the greater its peroxidation susceptibility. Combining the knowledge of the fatty acid composition of membrane lipids with these different susceptibilities one can calculate a single number, the peroxidation index (PI), that represents the membrane's susceptibility to lipid peroxidation [9].

Shmookler Reis *et al.* have measured the fatty acid composition of lipids extracted from strains of *Caenorhabditis elegans* that vary in longevity by ~10-fold, and report several significant log-linear correlations between longevity and fatty acid composition. The results are strongly influenced by two mutant strains (*daf-2* and *age-1*) that show the greatest longevities. Their findings can be summarized thus; comparing the shortest-living to longest-living strains total monounsaturates increased from 34% to 48%, total polyunsaturates decreased from 37% to 26%, and PI decreased from 141 to 81. The main influence on PI was the most polyunsaturated fatty acid (20:5n-3) which decreased from 14% to 6%. These changes are remarkably similar to correlations between PI and MLSP reported previously for mammals and birds. I have shown PI is inversely proportional to the ~0.3 power of MLSP [10]. A similar log-log analysis of the Shmookler Reis *et al.* data shows the PI of *C elegans* strains to also be inversely proportional to ~0.3 power of relative longevity. This similarity is intriguing. Another way of expressing this relationship is that longevity is inversely proportional to the cube power of PI. As Shmookler Reis *et al.* say there is no special reason why the relationship should be linear. Indeed we should expect a non-linear relationship if membrane fatty acid composition is influential because of the chain-reaction positive-feedback effects of lipid peroxidation [9]. Expressed another way: an ~20% decrease in the peroxidative susceptibility of membrane lipids is associated with a doubling (100% increase) of longevity.

Shmookler Reis *et al.* have measured total lipids and not specifically membrane lipids [4], yet because previous studies of *C. elegans* show polyunsaturates are predominantly located in phospholipids and not triacylglycerols [11] the changes in PI likely represent relative changes in membrane lipid composition. Previous studies have implicated lipid metabolism in nematode longevity but the importance of the Shmookler Reis *et al.* study is, in my opinion, that it is the first to concentrate on fatty acid composition (i.e. lipid quality) rather than lipogenesis and lipid quantity.

The emphasis on the importance of lipids for longevity as primarily a source of energy (adiposity) rather an essential component of membranes is, in my opinion, an unfortunate consequence of our approach to teaching biochemistry, which is generally anthropocentric rather than evolution-centric. We generally teach triacylglycerols (and energy-storage) first and phospholipids (and membrane structure) second. Yet from an evolutionary perspective phospholipids precede triacylglycerols. Our basic biochemistry, including lipogenesis, was evolved by prokaryotes and the best interpretation is that lipogenesis was an important process to manufacture membranes. All living organisms have lipid membranes like they possess DNA. Just as DNA has been described the ‘eternal molecule’, so membranes can be considered an ‘eternal structure’ as membranes are made from pre-existing membranes. Procaryotes do not store energy as triacylglycerol and the evolution of triacylglycerols as an efficient form of energy storage is largely a eukaryotic modification of the basic processes to make membrane lipids evolved by procaryotes. Indeed triacylglycerols are synthesised from a phospholipid (phosphatidic acid).

Shmookler Reis *et al.* have used various fatty acid ratios to get at the important enzymes possibly involved in extended longevity. They have also measured changes in transcript levels of a variety of elongase and desaturase genes and used RNAi knock-downs to examine the influence of these elongase and desaturase genes on both hydrogen peroxide resistance and longevity. For me the most interesting relate to the *fat-4* gene, which produces a desaturase that is essential for the manufacture of 20:5n-3. They show (i) from fatty acid ratios, 20:5n-3 is negatively correlated with longevity among the *C elegans* strains, (ii) *fat-4* transcript levels are reduced in F2 of the most longevous strain (*age-1*), and (iii) inhibition of this enzyme by RNAi knockdown produced both the greatest peroxide-resistance and longevity extension. Although Shmookler Reis *et al.* did not confirm that membrane fatty acid composition was changed by their treatments, earlier studies have shown that 20:5n-3 is absent in *fat-4* mutant *C. elegans* [12].

In conclusion, the paper by Shmookler Reis *et al.* represents a significant step towards our eventual understanding of the biochemical mechanisms that determine longevity.
